# Dexamethasone Enhances Achilles Tendon Healing in an Animal Injury Model, and the Effects Are Dependent on Dose, Administration Time, and Mechanical Loading Stimulation

**DOI:** 10.1177/03635465221077101

**Published:** 2022-03-02

**Authors:** Franciele Dietrich-Zagonel, Per Aspenberg, Pernilla Eliasson

**Affiliations:** † Department of Biomedical and Clinical Sciences, Faculty of Medicine and Health Science, Linköping University, Linköping, Sweden; Investigation performed at Linköping University, Linköping, Sweden

**Keywords:** corticosteroids, repair, resolution, rat, calcaneal tendon, biomechanics

## Abstract

**Background:**

Corticosteroid treatments such as dexamethasone are commonly used to treat tendinopathy but with mixed outcomes. Although this treatment can cause tendon rupture, it can also stimulate the tendon to heal. However, the mechanisms behind corticosteroid treatment during tendon healing are yet to be understood.

**Purpose:**

To comprehend when and how dexamethasone treatment can ameliorate injured tendons by using a rat model of Achilles tendon healing.

**Study Design:**

Controlled laboratory study.

**Methods:**

An overall 320 rats were used for a sequence of 6 experiments. We investigated whether the drug effect was time-, dose-, and load-dependent. Additionally, morphological data and drug administration routes were examined. Healing tendons were tested mechanically or used for histological examination 12 days after transection. Blood was collected for flow cytometry analysis in 1 experiment.

**Results:**

We found that the circadian rhythm and drug injection timing influenced the treatment outcome. Dexamethasone treatment at the right time point (days 7-11) and dose (0.1 mg/kg) significantly improved the material properties of the healing tendon, while the adverse effects were reduced. Local dexamethasone treatment did not lead to increased peak stress, but it triggered systemic granulocytosis and lymphopenia. Mechanical loading (full or moderate) is essential for the positive effects of dexamethasone, as complete unloading leads to the absence of improvements.

**Conclusion:**

We conclude that dexamethasone treatment to improve Achilles tendon healing is dose- and time-dependent, and positive effects are perceived even in a partly unloaded condition.

**Clinical Relevance:**

These findings are promising from a clinical perspective, as the positive effect of this drug was seen even when given at lower doses and in a moderate loading condition, which better mimics the load level in patients with tendon ruptures.

Corticosteroids are widely used in the clinic to treat chronic tendon disorders.^13,36^ However, this treatment might be detrimental because of adverse side effects and may even cause tendon ruptures.^29,31^ Corticosteroids can be administered as systemic or local treatment, and this might have diverse immunological effects and hence modulate tendon healing. Local injections are controversial^41^ and show negative^28,42^ and positive^35^ effects on intact and healing tendons. We previously showed in rats that systemic corticosteroid treatment during the early inflammatory phase (days 0-4) impaired tendon healing.^6^ In contrast, an improvement of the material properties of the tendon was seen when the drug was administered during the proliferative phase of healing (days 5-9).^6^ Nevertheless, when and how this drug should be administered for optimal healing needs to be better understood.

The Achilles tendon builds the connection between the calf muscles (gastrocnemius and soleus) and the calcaneal bone.^12^ This tendon is mainly formed by collagen,^14^ and even though if it can stretch, injuries are common. Healing of tendon injuries is usually divided into 3 overlapping phases.^30,43^ In rats, the healing process occurs faster than in humans,^11,20^ and the exact days that each phase starts and finishes depend on many factors, such as the size of the injury. The inflammatory phase starts at the time of the injury and persists for some days, while the proliferative and remodeling phases endure for longer periods. After an injury, inflammation has to be resolved for regeneration to start.^6,40^ Anti-inflammatory drugs such as dexamethasone act through resolution of the inflammation.^1,3,45^ We hypothesized that delayed dexamethasone treatment would lead to faster resolution, earlier remodeling, and enhanced mechanical properties of the healing tendon.

Many factors are known to influence the tendon-healing process. The circadian rhythm has been reported to regulate collagen homeostasis in intact tendons.^8^ Although the circadian rhythm probably influences tendon healing, little is known about this. Changes in the microbiome have been reported to influence tendon healing as well as different immunomodulatory treatments, including platelet-rich plasma and corticosteroids.^15,17^ Moreover, there are interactions between the effect of loading and immunological changes during tendon healing.^16^ Different load magnitudes have been shown to activate distinct mechanisms and have diverse effects on the structural and material properties of the healing tendon.^16,23^ Full loading triggers a stronger proinflammatory response than moderate loading, possibly because of microdamage and infiltrating leukocytes.^5,22^ Despite this obvious effect on the inflammatory response, previous studies on the effect of dexamethasone on tendon healing have used full loading models,^6,17^ and different load levels might display distinct outcomes. Hence, a more comprehensive understanding is essential in terms of how this treatment interacts with the immune system and how it responds when having altered load magnitudes, especially because patients with tendon ruptures seldom have high-load magnitudes on their injured tendons.

Our study was based on a sequence of experiments. Every new finding led us to a new hypothesis and a new research question. The aim of this study was (1) to find the optimal administration time, route, and dose of dexamethasone for improving Achilles tendon healing and (2) to investigate if the positive effect of this treatment depends on the level of mechanical loading.

## Methods

### Study Design

Specific pathogen–free female Sprague-Dawley rats were used (n = 320; Taconic Biosciences). The study was performed as a sequence of 6 experiments. Each group consisted of 10 randomly assigned rats (by lottery), except for the groups used for flow cytometric analysis and histological analysis (n = 6 in each group). All animals were euthanized 12 days postoperatively. Experiments were approved by the regional ethics committee for animal experiments in Linköping (15-15 and 1424).

#### Experiments 1-3

The aim of experiments 1, 2, and 3 was to study if different levels and injection time points for dexamethasone treatment resulted in diverse effects on tendon healing (Table 1). Dexamethasone (Dexaject; Dopharma Research BV) was given at a dose of 0.5 or 0.1 mg/kg for 5 or 2 consecutive days or as a single injection. Experiment 1 was unintentionally performed when the light cycle was reversed, and injections were performed at 11 AM. This experiment was repeated with injections at 7 AM because our positive control (dexamethasone; 0.5 mg/kg; given at days 5 to 9) had an unexpectedly small effect as compared with previous data.^6,17^ Experiments 2 and 3 were performed with a standard light cycle and injections at 3.30 PM. Saline solution 0.9% (B Braun Melsungen AG) was given to the control groups.

**Table 1:** Experimental Setup With the 6 Experiments^*a*^

	Day											
	-4	0^*b*^	4	5	6	7	8	9	10	11	12^*c*^	No.
**Experiment 1**												
11 AM												
Saline				NaCl	NaCl	NaCl	NaCl	NaCl				10
Dexa × 5				0.5	0.5	0.5	0.5	0.5				10
Dexa × 5				0.1	0.1	0.1	0.1	0.1				10
Dexa × 1				0.5								10
7 AM												
Saline				NaCl	NaCl	NaCl	NaCl	NaCl				10
Dexa × 5				0.5	0.5	0.5	0.5	0.5				10
Dexa × 5				0.1	0.1	0.1	0.1	0.1				10
Dexa × 2				0.5	0.5							10
Dexa × 1				0.5								10
**Experiment 2**												
Saline				NaCl	NaCl	NaCl	NaCl	NaCl				10
Dexa 4-8			0.5	0.5	0.5	0.5	0.5					10
Dexa 5-9				0.5	0.5	0.5	0.5	0.5				10
Dexa 6-10					0.5	0.5	0.5	0.5	0.5			10
Dexa 7-11						0.5	0.5	0.5	0.5	0.5		10
**Experiment 3**												
Saline						NaCl	NaCl	NaCl	NaCl	NaCl		10
Dexa 0.5 × 1						0.5						10
Dexa 0.5 × 2						0.5	0.5					10
Dexa 0.5 × 5						0.5	0.5	0.5	0.5	0.5		10
Dexa 0.1 × 5						0.1	0.1	0.1	0.1	0.1		10
**Experiment 4**												
Saline local						NaCl	NaCl	NaCl	NaCl	NaCl		10
Dexa local, 0.1						0.1	0.1	0.1	0.1	0.1		10
Dexa local, 0.02						0.02	0.02	0.02	0.02	0.02		10
Saline systemic						NaCl	NaCl	NaCl	NaCl	NaCl		6
Dexa systemic, 0.1						0.1	0.1	0.1	0.1	0.1		6
Dexa local, 0.1						0.1	0.1	0.1	0.1	0.1		6
**Experiment 5**												
Saline						NaCl	NaCl	NaCl	NaCl	NaCl		10
Dexa						0.1	0.1	0.1	0.1	0.1		10
Late mod. loading						Botox						10
**Experiment 6**												
Saline, full loading						NaCl	NaCl	NaCl	NaCl	NaCl		16
Dexa, full loading						0.1	0.1	0.1	0.1	0.1		16
Saline, moderately loaded	Botox					NaCl	NaCl	NaCl	NaCl	NaCl		10
Dexa, moderately loaded	Botox					0.1	0.1	0.1	0.1	0.1		10
Saline, unloaded	Botox	Boot^*d*^				NaCl	NaCl	NaCl	NaCl	NaCl		10
Dexa, unloaded	Botox	Boot^*d*^				0.1	0.1	0.1	0.1	0.1		10

a Doses: 0.5, 0.1, and 0.02 mg/kg. All experiments except experiment 1 had injections at 3:30 PM and a standard light cycle in the room. Dexa, dexamethasone.

b Tendon transection.

c Euthanization.

d Steel orthosis.

#### Experiment 4

The aim of experiment 4 was to compare systemic and local administration routes for dexamethasone. Dexamethasone was given as local (0.1 or 0.02 mg/kg) or systemic (0.1 mg/kg) injections for 5 consecutive days (days 7-11). Local injections were performed with an insulin syringe. Saline was also given as local injections.

#### Experiment 5

Experiments 1 to 3 showed that high doses of dexamethasone led to reduced muscle weight. As such, the aim of experiment 5 was to investigate if the effect of dexamethasone derived from a delayed reduction in loading (attributed to less muscle mass) or from a drug-specific effect. One group was therefore given injections of botulinum toxin (Botox) in the calf muscle at day 7 to achieve a delayed reduction in loading. This group was compared with dexamethasone treatment and saline.

#### Experiment 6

The aim of experiment 6 was to investigate if the positive effect of dexamethasone treatment (0.1 mg/kg) depends on the level of tensile loading. Dexamethasone or saline was administered to rats with full loading (free cage activity), moderate loading (Botox injections in the calf muscle), or complete unloading (Botox injections in the calf muscle and a steel orthosis boot) for 5 consecutive days.

### Standard Procedures

#### Animals

Female rats weighing on average 213 g (SD, 18 g) were placed in pairs into acrylic cages containing wooden pegs, shredded paper, and hiding places. The cages were individually ventilated, and the room was kept at a controlled temperature of 22°C, humidity of 55%, and a 12-hour light-dark cycle. A standard light cycle means light from 7 AM to 7 PM. Food and water were given ad libitum.

#### Model Used to Reduce Loading

**Figure 1.**
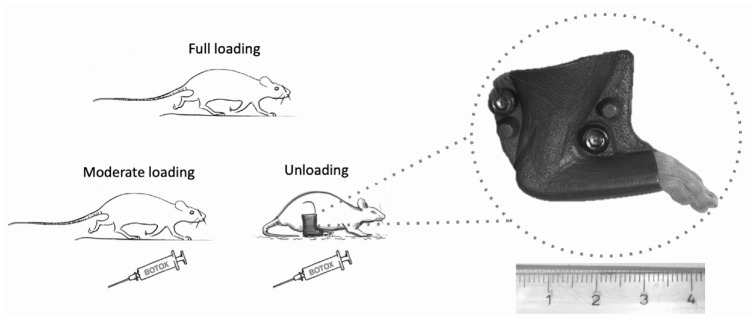
Image of the loading modalities used in experiments 5 and 6. Moderate loading was achieved by Botox injections into the calf muscle, and complete unloading was achieved by combining Botox treatment with a steel orthosis boot to restrict ankle joint motion. Ruler measurement is in cm.

Botox injections were used to reduce tensile loading (moderate loading) (Table 1, Figure 1). Botox (Allergan) injections were performed under anesthesia with isoflurane gas (Forene; Abbot Scandinavia). The gastrocnemius lateralis, gastrocnemius medialis, and soleus muscles in the right leg were injected with 1 U of Botox per muscle, for a total of 3 U and 0.06 mL per animal. A steel orthosis boot (Prodelox) was used in the unloaded group after tendon surgery to prevent joint movement and passive loading, accomplishing complete unloading. Botox effectiveness was visually confirmed before surgery.

#### Surgical Procedure

Complete tendon transection was achieved under general anesthesia with isoflurane gas (Forene; Abbot Scandinavia) under aseptic conditions. Subcutaneous antibiotic (25 mg/kg, oxytetracycline; Emgemycin [Intervet]) was given once preoperatively, and subcutaneous analgesia (0.045 mg/kg, buprenorphine; Temgesic [Indivior Europe Limited]) was given pre- and postoperatively. During surgery, rats were placed in a prone position. A minor skin incision was made lateral to the right Achilles tendon to expose the tendon complex. The plantaris tendon was removed and the Achilles tendon was completely transected with a single transversal cut in the midtendon portion. The tendon was left to heal nonsutured, and the skin was closed.

#### Mechanical Testing

Ten rats in each group were euthanized with carbon dioxide 12 days postsurgery, and the tendon was harvested in a standardized way with the calcaneal bone and calf muscle. The transverse area and gap length were measured by a caliper. These measurements were performed twice on a subset of the tendons (n = 50) by the same investigator (F.D.Z.) (*P* < .001, *R*^2^ = 0.96, for transverse area; *P* < .001, *R*^2^ = 0.94, for gap length). The samples were thereafter weighted before the majority of the muscle was scraped out. The tendon was mounted in the materials testing machine (100R; DDL Inc) and pulled at a constant speed of 0.1 mm/s until failure. Peak force at failure (N) and energy uptake until failure (N/mm) were recorded by the software (MtestW Version 5.1.0; ADMET). The investigator marked a linear portion of the curve for automated stiffness calculation (N/mm). Peak stress (MPa; peak force/transverse area) and estimation of elastic modulus (MPa; stiffness × gap length/transverse area) were calculated assuming an elliptical cylindrical shape and homogeneous mechanical properties. The method used in this study has been described previously.^17^ All surgical procedures and mechanical tests were performed blinded from treatment by giving the tendons a random identification number before they were measured and tested.

#### Flow Cytometry

Eighteen rats were used for immune cell characterization by flow cytometric analyses after local and systemic dexamethasone treatment or saline. The analysis was performed to investigate if local injections gave a systemic response. Rats were anesthetized with isoflurane gas. Blood was collected by a cardiac puncture and immediately placed into tubes containing EDTA (BD Vacutainer) and kept on ice. To separate the mononuclear cells, the blood was carefully layered on Histopaque-1119 (Sigma-Aldrich) and centrifuged at room temperature (700*g* for 45 min), followed by buffy coat collection and addition of support buffer (RPMI 1640 without l-glutamine and phenol red, 4% inactivated fetal bovine serum, 5 mM EDTA, and 25 mM HEPES). The suspension was washed twice at 600*g* for 6 minutes, and 1 to 3 million cells were collected in Cell Staining Buffer (Biolegend) and incubated 20 minutes while protected from the light and on ice with antibodies (Appendix Table A1, available in the online version of this article). For live/dead discrimination, Zombie Violet (Biolegend) was added. Cells were fixed in 2% paraformaldehyde at room temperature (Biolegend) and washed twice with Cell Staining Buffer. Cells from a control rat were used for fluorescence minus one gating. To sustain blinding, the operator (F.D.Z.) did not know which rat this was. FACS Aria III (BD Biosciences) was used in this study, and Cytometer Setup and Tracking Beads (BD Biosciences) ensured the stability of the cytometer. Compensation was performed with the same antibodies as in the experiment, and the gatings of discrete antigens were set on population morphology. In all samples, initial gating was performed on singlet cells, scatter parameters, and live cells to define single living leukocytes. Gating was performed in FlowJo Version 10.0.7 (Treestar). This method has been described in detail previously.^4^

#### Histological Examination

Twelve rats were used for histological imaging with normal hematoxylin and eosin staining on saline- and dexamethasone-treated tendons. Rats were anesthetized with isoflurane gas at 12 days postsurgery. Tendons were harvested and fixed in 4% phosphate-buffered formaldehyde overnight, followed by dehydration and paraffin embedding. Longitudinal sections (7 µM) were made, and hematoxylin and eosin staining was performed. Images were captured using a light microscope (Olympus BX51) with an attached camera (Olympus DP73) and the software cellSens Entry (Version 1.8.1; Olympus Corporation). Three objective lenses were used: 4×/0.13, 10×/0.30, and 20×/0.50 (UPlanFL; Olympus).

#### Exclusion

One rat in the dexamethasone 0.1-mg/kg group in experiment 1 was excluded from the mechanical analysis because of rupture by the distal clamp. One rat in the dexamethasone ×1 group in experiment 3 was excluded from analysis owing to rupture by the proximal clamp. Blood collection for flow cytometry analysis in experiment 4 failed in 4 rats in the local treatment group. Two rats were also excluded from the mechanical analysis in experiment 4, 1 in the saline group and 1 in the dexamethasone systemic group, owing to rupture by the clamp.

### Statistical Analysis

The results were analyzed using SPSS Version 21 (IBM), and graphs were created using Prism Version 9 (GraphPad). The predefined primary variable was always peak stress. Experiments 1 to 4 were analyzed by independent-samples *t* tests. Experiment 5 was analyzed with 1-way analysis of variance, followed by Bonferroni post hoc for multiple-comparison analyses, and experiment 6 was analyzed by a 2-way analysis of variance. Independent *t* tests comparing the treated and saline groups were performed within each loading condition.

## Results

### Experiment 1

The positive effect of dexamethasone is dependent on the administration time point during the day, possibly through the circadian rhythm.

**Figure 2.**
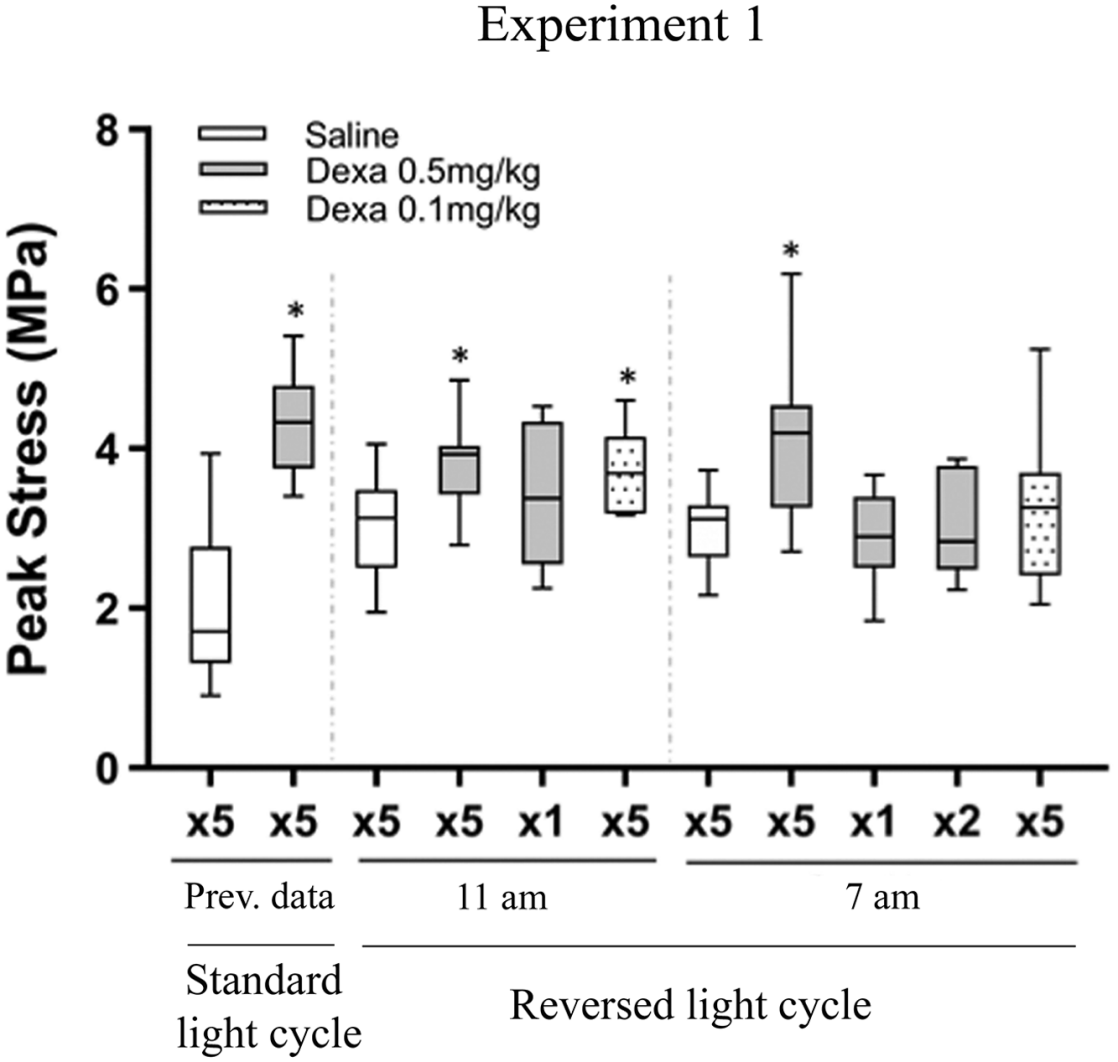
Peak stress from experiment 1. The experiment was performed with 2 repetitions, both with a reversed light cycle in the room. Injections were done at 11 or 7 AM, and 2 dexamethasone (Dexa) doses were used: 0.5 mg/kg (gray boxes) and 0.1 mg/kg (dotted boxes). Saline was used as control. Injections were performed for 5 or 2 consecutive days (×5 or ×2) or as a single injection (×1). *Significant difference vs saline. The peak stress was increased in Dexa ×5 but less when compared with previous data performed under a standard light cycle.^6^ Line, median; box, interquartile range; error bars, minimum and maximum.

Experiment 1 showed that dexamethasone treatment (0.5 mg/kg) increased the peak stress of the tendon by 27% (*P* = .008) and elastic modulus by 70% (*P* < .001) (Appendix Table A2, available online). The effect on peak stress was, however, not as powerful as previously observed^6^ (Figure 2). The experiment was repeated with similar results. The discrepancy in the magnitude of increase in peak stress between experiment 1 and previous data was then traced to the reversed light cycle, as all previous studies had been performed with a standard light cycle. Experiment 1 also showed that dexamethasone treatment led to an increased gap length and a smaller transverse area as compared with saline (*P* < .05 for both).

### Experiment 2

Treatment delay into the later healing phase gives further improvement of the structural and material properties of the tendon.

**Figure 3.**
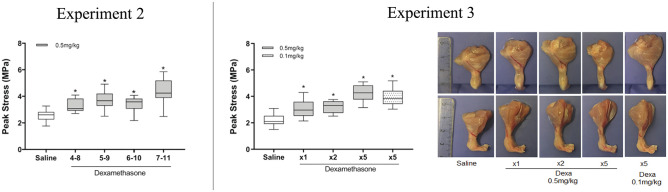
Peak stress and gross tendon morphology from experiments 2 and 3. The highest peak stress in experiment 2 was seen in the group that received dexamethasone (Dexa) treatment between days 7 and 11. The peak stress in experiment 3 increased irrespective of Dexa dose and number of injections when treatment was initiated at day 7. Gross morphology of the Achilles tendon 12 days after the injury, with and without Dexa treatment (days 7-11). Note that in the top row, images were taken with a dorsal view while in the bottom row it is the same tendon but with a lateral view. All images were taken with a standardized height. *Significant difference vs saline. Line, median; box, interquartile range; error bars, minimum and maximum.

Experiment 2 showed that dexamethasone treatment, irrespective of treatment initiation, led to increased material properties, with an increase in peak stress and estimate of elastic modulus (*P* < .005 for all groups vs saline). The peak stress was 19% higher in the dexamethasone days 7-11 group as compared with our positive control (dexamethasone, days 5-9), although this difference was not statistically significant (Table 2, Figure 3). However, peak force was significantly higher in the dexamethasone days 7-11 group versus the saline group and dexamethasone days 5-9 group. Stiffness was higher in the dexamethasone days 6-10 group and days 7-11 group than in the saline group. Experiment 2 showed once more that 0.5 mg/kg of dexamethasone resulted in adverse effects, as measured by a smaller transverse area and calf muscle atrophy (*P* < .05 for both). The gap length in the dexamethasone days 7-11 group was similar to that in the saline group but in contrast to the dexamethasone days 5-9 group.

**Table 2:** Mechanical Data From Experiment 2^*a*^

	Saline	Dexa 5-9	*P* Value	%	Dexa 4-8	*P* Value	%	Dexa 6-10	*P* Value	%	Dexa 7-11	*P* Value	%
Material properties	2.5 (0.4)	3.7 (0.7)	**<.001**	48	3.3 (0.5)	**.002**	32	3.4 (0.6)	**.002**	36	4.4 (1)	**<.001**	76
Peak stress, MPa	3.1 (0.8)	6 (0.9)	**<.001**	94	6 (1)	**<.001**	94	5.2 (1.5)	**.001**	68	5.4 (1.9)	**.002**	74
Est. elastic modulus, MPa													
Structural properties													
Transverse area, mm^2^	14 (2.1)	9.9 (2)	**<.001**	−29	9.4 (1.2)	**<.001**	−33	12 (3.1)	**.033**	−14	11 (2.4)	**.003**	−21
Gap length, mm	7.8 (0.8)	9.3 (1.1)	**.002**	19	9.8 (1.2)	**<.001**	26	8.5 (1.2)	.149	9	8.0 (1.3)^*b*^	.639	3
Peak force, N	35 (3.4)	37 (10)	.629	6	31 (5.8)	.055	−11	39 (12)	.379	11	47 (11)^*b*^	**.004**	34
Stiffness, N/mm	5.5 (0.6)	6.5 (1.8)	.132	18	5.4 (1.2)	.765	−2	6.8 (1.9)	**.047**	24	7.0 (1.2)	**.003**	27
Energy uptake, N/mm	86 (15)	70 (22)	**.074**	−19	67 (7.9)	**.002**	−22	78 (29)	.443	−9	103 (30)^*b*^	.122	20
Sample weight, g	2.3 (0.3)	1.8 (0.1)	**<.001**	−22	1.9 (0.2)	**.005**	−17	1.8 (0.2)	**<.001**	−22	1.9 (0.3)	**.007**	−17

a Values are presented as mean (SD). Percentage was calculated in relation to the saline group (n = 10 in each group). Bold indicates significant difference vs saline. Dexa, dexamethasone.

b Significant difference vs positive control (Dexa, 5-9).

### Experiment 3

When using the optimal time point (days 7-11), dexamethasone dose can be reduced 5-fold and still enhance tendon healing.

Experiment 3 showed that dexamethasone treatment increased peak stress in all groups as compared with saline. The most pronounced effect was seen with 5 consecutive injections of dose 0.5 mg/kg (95% increase) or 0.1 mg/kg (77% increase), with no statistical difference between them (*P* = .29) (Table 3, Figure 3). Elastic modulus was increased by 169% and 142%, respectively. The transverse area was reduced after 5 injections (irrespective of the dose) or 2 injections but not after a single injection. Muscle atrophy was seen in all treated groups but less pronounced in the groups with 0.1 mg/kg or a reduced number of injections. Gap length was increased in all dexamethasone-treated tendons as compared with saline, while stiffness was increased after 5 injections, irrespective of the dose.

**Table 3:** Mechanical Data From Experiment 3^*a*^

	Saline	Dexa ×5, 0.5 mg/kg	*P* Value	%	Dexa ×5, 0.1 mg/kg	*P* Value	%	Dexa ×1, 0.5 mg/kg	*P* Value	%	Dexa ×2, 0.5 mg/kg	*P* Value	%
Material properties													
Peak stress, MPa	2.2 (0.5)	4.3 (0.6)	**<.001**	95	3.9 (0.7)	**<.001**	77	3.1 (0.7)^*b*^	**.007**	41	3.2 (0.4)^*b*^	**<.001**	45
Est. elastic modulus, MPa	2.6 (0.8)	7.0 (1.7)	**<.001**	169	6.3 (1.6)	**<.001**	142	4.3 (1.4)^*b*^	**.005**	65	4.4 (1.2)^*b*^	**.001**	69
Structural properties													
Transverse area, mm^2^	14 (2.9)	8.6 (1.5)	**<.001**	−39	9.5 (1.7)	**<.001**	−32	12 (1.5)^*b*^	.081	−14	11 (1.2)^*b*^	**.013**	−21
Gap length, mm	6.8 (1.1)	8.8 (1.1)	**.001**	29	8.3 (0.9)	**.003**	22	8.2 (0.9)	**.006**	21	8.5 (0.9)	**.001**	25
Peak force, N	31 (6.9)	37 (8.2)	.120	19	37 (5.9)	.062	19	37 (6)	.075	19	37 (4.6)	**.037**	19
Stiffness, N/mm	5.5 (1.4)	6.8 (1.2)	**.044**	24	7.1 (1.9)	**.039**	29	6.2 (1.2)	.239	13	5.8 (1)	.497	5
Energy uptake, N/mm	69 (14)	65 (22)	.589	−6	73 (14)	.526	6	80 (13)	.104	16	76 (15)	.296	10
Sample weight, g	2.3 (0.3)	1.7 (0.1)	**<.001**	−26	2 (0.2)^*b*^	**.020**	−13	1.9 (0.4)	**.035**	−17	1.9 (0.2)^*b*^	**.010**	−17

a Values are presented as mean (SD). Percentage was calculated in relation to the saline group (n = 10 in each group except for dexamethasone (Dexa) ×1, n = 9). All injections were given from day 7 to day 11. Bold indicates significant difference vs saline.

b Significant difference vs 5 injections with Dexa, 0.5 mg/kg.

### Experiment 4

The positive dexamethasone outcomes on healing tendons are reliant on the route of administration, although local treatment still triggers granulocytosis and T-cell reduction.

**Figure 4.**
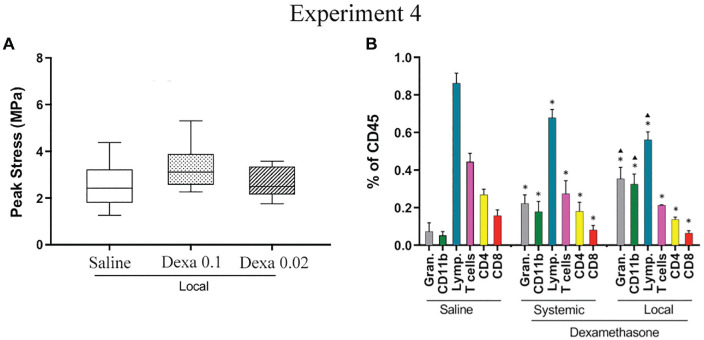
Mechanical and flow cytometry data after local and systemic dexamethasone (Dexa) treatment for 5 consecutive days. (A) Peak stress after local treatment on days 7 to 11 with saline (n = 9) or Dexa (0.1 mg/kg, n = 9, dotted boxes; 0.02 mg/kg, n = 10, striped boxes). Saline, n = 9; Dexa, n = 9 (0.1 mg/kg) and n = 10 (0.02 mg/kg). Line, median; box, interquartile range; error bars, minimum and maximum. (B) Flow cytometry data from animals receiving Dexa (systemic or local injections) or saline (systemic) on days 7 to 11. Saline, n = 6; systemic Dexa, n = 6; local Dexa , n = 2. Gran, granulocytes; lymp, lymphocytes. *Significant difference vs saline. ▢Significant difference vs Dexa systemic. Bar, mean; error bars, SD.

Experiment 4 showed that peak stress did not differ between the groups with local dexamethasone treatment and local saline treatment (Table 4, Figure 4A). Dexamethasone-treated tendons (0.1 mg/kg) became slightly stiffer when compared with saline. Flow cytometric analysis of the peripheral blood showed a systemic reaction to the dexamethasone despite local treatment. Dexamethasone-treated animals, local and systemic, showed signs of granulocytosis, with a specific increase in the CD11b population as well as lymphopenia (Figure 4B; Appendix Table A4, available online). The effect on granulocytes, CD11b subpopulation, and lymphocytes was surprisingly somewhat more pronounced in the locally treated animals in relation to the systemic ones (*P* < .05). Dexamethasone, independent of the administration route, also induced a small reduction in CD8+ and CD4+ T-cell populations (*P* < .05).

**Table 4:** Mechanical Results From Experiment 4^*a*^

	Saline	Dexa, 0.1 mg/kg	*P* Value	%	Dexa, 0.02 mg/kg	*P* Value	%
Material properties							
Peak stress, MPa	2.6 (1.0)	3.2 (0.9)	.146	23	2.7 (0.7)	.787	4
Est. elastic modulus, MPa	2.8 (1.3)	3.5 (1.0)	.192	25	2.8 (1.2)	.972	0
Structural properties							
Transverse area, mm^2^	15 (3.8)	14 (3.0)	.296	−7	16 (2.7)	.935	0
Gap length, mm	7.9 (1.1)	7.7 (1.4)	.747	−3	7.2 (1.2)	.278	−9
Peak force, N	37 (7.5)	44 (13)	.173	19	40 (7.4)	.318	8
Stiffness, N/mm	4.9 (0.8)	6.4 (1.9)	**.048**	31	5.7 (1.2)	.124	16
Energy uptake, N/mm	94 (32)	94 (30)	.985	0	90 (27)	.741	−4
Sample weight, g	2.1 (0.2)	1.9 (0.2)	.061	−10	1.9 (0.2)	**.050**	−10
Muscle weight, g	1.6 (0.2)	1.4 (0.2)	.099	−13	1.4 (0.2)	.076	−13

a Values are presented as mean (SD). Percentage was calculated in relation of the saline group (n = 9 for saline and dexamethasone [Dexa], 0.1 mg/kg; n = 10, Dexa, 0.02 mg/kg). Bold indicates significant difference vs saline.

### Experiment 5

Reduced loading by paralyzing the calf muscle does not mimic the dexamethasone effect.

To differentiate between reduced loading attributed to muscle atrophy and the treatment effect, a comparison was done between dexamethasone treatment and delayed load reduction by Botox injections in experiment 5. Dexamethasone treatment resulted in improved tendon material proprieties, while delayed load reduction reduced all analyzed tendon mechanical parameters (Appendix Table A3, available online).

### Experiment 6

The effect of dexamethasone on tendon mechanical properties differs by load magnitude.

**Figure 5.**
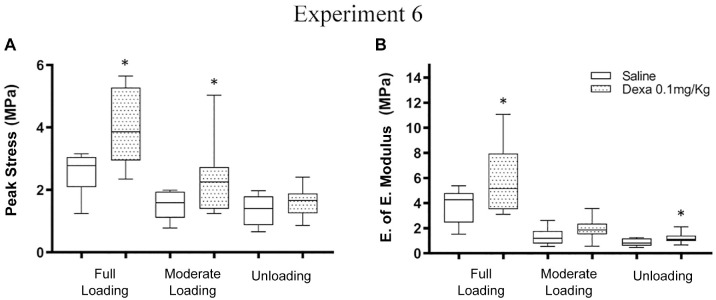
Material properties from experiment 6. (A) Peak stress. (B) Estimation of elastic (E. of E.) modulus. All groups received daily systemic injections of saline or dexamethasone ([Dexa] 0.1 mg/kg; days 7-11). All groups, n = 10. *Significant difference vs saline. Line, median; box, interquartile range; error bars, minimum and maximum.

**Figure 6.**
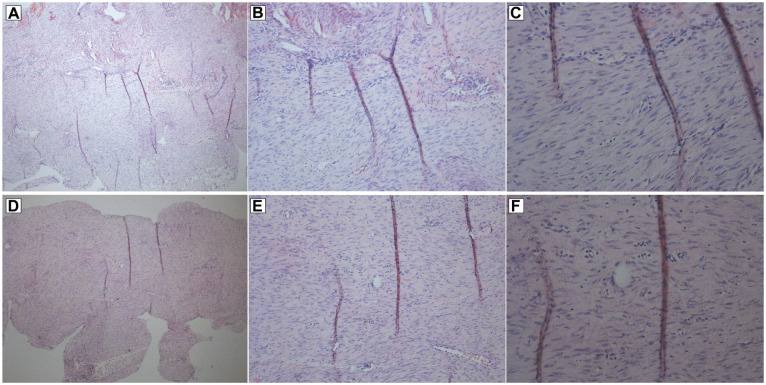
Hematoxylin and eosin staining of tendons from fully loaded rats treated with (A-C) saline or (D-F) 0.1 mg/kg of dexamethasone on days 7 to 11. Magnification for images: (A, D) 40×, (B, E) 100×, (E, F) 200×.

Experiment 6 showed no significant interaction between loading and dexamethasone treatment for our predefined variable peak stress (*P* = .09) (Table 5). Dexamethasone treatment had positive effects in all loading conditions, although the effect was more pronounced with increased loading. Dexamethasone treatment increased peak stress by 50% to 60% when compared with saline in fully and moderately loaded groups but not in the unloaded group (Figure 5). Stiffness was increased in the moderately loaded group by approximately 50% (*P* < .04). Moreover, the transverse area tended to be smaller in the fully loaded group (*P* = .053) but not in the other groups. Muscle weight was significantly reduced by dexamethasone treatment in the unloaded group, although not in the other loading conditions. Hematoxylin and eosin staining of fully loaded rats (saline and dexamethasone) revealed no distinct difference between the groups with regard to cellularity or matrix structure (Figure 6).

**Table 5:** Mechanical Results From Experiment 6^*a*^

	Full Loading				Moderate Loading				Unloading				2-Way ANOVA, *P* Value		
	Saline	Dexa	*P* Value	%	Saline	Dexa	*P* Value	%	Saline	Dexa	*P* Value	%	Interaction	Treatment	Loading
Material properties															
Peak stress, MPa	2.6 (0.7)	4.0 (1.2)	**.004**	54	1.5 (0.4)	2.4 (1.1)	**.036**	60	1.3 (0.5)	1.6 (0.5)	.184	23	.087	**<.001**	**<.001**
Est. elastic modulus, MPa	3.9 (1.3)	5.9 (2.6)	**.046**	51	1.3 (0.7)	1.9 (0.8)	.090	46	0.9 (0.3)	1.2 (0.4)	**.043**	33	.105	**.005**	**<.001**
Structural properties															
Transverse area, mm^2^	12 (3.1)	9.2 (2.0)	.053	−23	8.6 (1.8)	7.7 (1.1)	.216	−10	8.8 (1.1)	7.9 (1.6)	.181	−10	.336	**.007**	**<.001**
Gap length, mm	8.3 (1.0)	7.9 (1.6)	.565	−5	2.8 (0.9)	2.6 (1.0)	.641	−7	2.2 (0.7)	2.1 (0.5)	.700	−5	.927	.404	**<.001**
Peak force, N	29 (9.4)	35 (7.2)	.123	21	13 (4.4)	18 (6.8)	.069	38	12 (4.0)	13 (3.5)	.601	8	.393	**.017**	**<.001**
Stiffness, N/mm	5.1 (1.5)	6.4 (1.4)	.063	25	4.0 (1.6)	5.9 (2.1)	**.040**	48	3.6 (1.6)	4.5 (1.1)	.205	25	.588	**.002**	**.004**
Energy uptake, N/mm	69 (20)	71 (15)	.779	3	14 (4.9)	20 (8.2)	.099	43	13 (3.9)	13 (4.7)	.915	0	.767	.373	**<.001**
Sample weight, g	2.1 (0.3)	1.8 (0.2)	**.037**	−14	1.2 (0.2)	1.1 (0.2)	.058	−8	1.1 (0.1)	0.8 (0.1)	**<.001**	−27	.821	**<.001**	**<.001**
Muscle weight, g	1.5 (0.2)	1.4 (0.2)	.072	−7	0.8 (0.2)	0.7 (0.1)	.086	−13	0.7 (0.1)	0.6 (0.1)	**.001**	−14	.802	**<.001**	**<.001**

a Values are presented as mean (SD). Percentage was calculated in relation to the saline group (n = 10 in each group except for the muscle weight measurement for the saline unloading group, n = 8). Bold indicates significant difference vs saline. ANOVA, analysis of variance; Dexa, dexamethasone.

## Discussion

With this study, not only did we confirm that dexamethasone treatment can ameliorate Achilles tendon healing,^6,17^ but we also showed a more pronounced improvement in the material properties when treatment was protruded into the proliferative/early remodeling phase and that the positive effect remained when the daily loading was reduced. Furthermore, despite a significant reduction in the dexamethasone dose, the beneficial effects of the drug were still observed, making our findings more relevant in a clinical perspective. Additionally, dexamethasone, independent of the administration route, led to alterations in the immune system on a systemic level. Moreover, the positive effects of dexamethasone seem to be affected by the circadian rhythm.

The circadian rhythm is important for maintaining intact tendon tissue function and organization, and it influences collagen homeostasis.^8^ We observed that our initial experiments led to unexpectedly low improvement in our positive control. We were able to trace this back to the change in light cycle in the animal facility. The altered light cycle led to changes in the timing of the injections, from evening to morning. Previous studies have shown that disruptions in the circadian rhythm can lead to irregular fibril structures and a reduction in maximum load and elastic modulus in intact Achilles tendons.^8^ Furthermore, rat cornea mitotic activity in response to dexamethasone treatment has been reported to differ according to the time of the day when the drug is administred.^7^ Previous studies on healing tendons and dexamethasone treatment were all performed with injections done a few hours before the rats woke up.^6,17^ However, when the light cycle was altered to a reversed light cycle, injections were instead given in the morning when rats were starting to be active.^18^ In addition, although the rats in our study had been acclimatized for 2 weeks, changes in the natural cycle can lead to stress, elevated cortisol levels, and altered wound healing.^9,19^ Earlier research has shown that higher cortisol levels can delay healing processes by modified cytokine production,^9^ leading to ongoing inflammation instead of proceeding into the proliferative/remodeling phase.

With this in mind, a normal light cycle with a standardized injection time was used in the remaining experiments (2-6). It is believed that inflammation should be resolved before tissue regeneration can start,^6,40^ and dexamethasone treatment for a short period may promote this. As seen previously, dexamethasone treatment during the proliferative phase (days 5-9) improves the material and structural properties of the healing tendon.^6,17^ Despite improved material properties in the tendons, we sometimes observed that treated tendons became elongated.^17^ Within this study, when the treatment was delayed further into the proliferative phase or perhaps into the early remodeling phase at days 7 to 11, more pronounced improvement of the material properties was seen. This delay in treatment also led to less tendon elongation when compared with our positive control. The presence of tendon elongation^38,39^ and fibrotic tissue^21^ after a tendon injury is a common clinical challenge. Better clinical results after an Achilles tendon rupture have been seen in patients with less elongation.^27^ An elongated tendon can hinder simple activities such as running and jumping; hence, an understanding of how to use dexamethasone treatment, without inducing tendon elongation is therefore essential.

High doses of dexamethasone might influence stress levels and result in adverse effects, such as reduced weight and consequently altered homeostasis.^26,32,33,37,44^ As such, it is important to investigate if a lower dose or a reduced number of injections could be used to improve tendon healing. A distinct healing response was seen according to the number of dexamethasone injections given, with peak stress varying by 41% to 95% (experiment 3), although better material properties were seen independent of the dose or number of injections. The strongest response was seen with the highest dose but with no significant difference when the dose was reduced 5-fold. Conversely, the higher dose did result in more pronounced muscle atrophy as compared with the lower dose or fewer injections. Tendon and muscle tissues have been reported to be influenced by sex hormones, especially estrogen, which has a role in skeletal muscle protein turnover.^24,25^ Female rats were used in all our experiments. The use of male rats could perhaps influence the effect of the drug on muscle and lead to different findings, but we have not investigated this. Furthermore, a reduction in load magnitude can lead to muscle atrophy.^2,16,20,23^ Mechanical loading profoundly affects tendon healing, and the dexamethasone effects found here could be, in reality, an effect of reduced loading levels. We did test this hypothesis by reducing the load from day 7, but this instead impaired all mechanical parameters, in contrast to the improvement seen with dexamethasone treatment.

Dexamethasone treatment had more pronounced effects in fully or moderately loaded rats than unloaded ones. The effect in fully loaded rats corresponds to that found in previous studies.^6,17^ However, full loading does not relate well to humans with an Achilles tendon injury; thus, moderate loading is more relevant. Full loading and moderate loading have been shown to activate somewhat different mechanisms after a tendon rupture. Full loading acts through mechanotransduction and microdamage and leads to an increased proinflammatory response, while moderate loading acts primarily through mechanotransduction.^23^ As a positive effect of dexamethasone also was observed with reduced loading, this might indicate that dexamethasone improves tendon healing in more ways than an immunomodulatory effect, possibly through a direct effect on tendon cells.^13^ Notably, moderate loading had no significant effect on muscle weight, as seen with full loading and unloading. The exact mechanism of corticosteroids during tendon healing is not yet clear, although one study did show a reduced number of CD8a+ cells in dexamethasone-treated tendons and improved collagen alignment.^6^

We observed that total granulocytes and the CD11b population increased, while CD4+ and CD8+ T cells decreased, after systemic and local dexamethasone treatment. Granulocytosis^34^ and lymphopenia^10^ are known effects of corticosteroids, and the results in the locally treated group indicate that the systemic effect cannot be avoided even with local injections. Local corticosteroid injections have been reported to cause detrimental effects on intact^42^ and healing^28^ tendons. Although we did not observe any adverse effects by local treatment, beneficial effects were absent. Overall, positive mechanical outcomes of dexamethasone on healing Achilles tendons seem to be reliant on the route of administration.

This study is not without limitations. We used only female rats, as they grow more slowly and are more reproducible in the mechanical testing. Furthermore, the same investigator performed all surgical procedures, injections, and mechanical tests. This can be considered a limitation or an advantage. The results in the experiments have probably less variation, but this can also include a bias. However, all surgery and mechanical testing were performed with the investigator blinded from treatment.

To the best of our knowledge, this study is the first to demonstrate that the beneficial effects of dexamethasone on tendon healing are dependent on the timing of treatment, route of drug administration, drug dose, and degree of loading. Additional studies on the mechanisms behind the effect of dexamethasone are desirable for further conclusions, as the treatment might act via systemic effects and local effects specific to the tendon cell and matrix. Moreover, the positive effects of corticosteroid treatment, without adverse effects, are more pronounced during the late proliferative phase or early remodeling phase (by daily systemic injections from days 7 to 11) and using the 0.1 mg/kg dose.

## Conclusion

Dexamethasone treatment can improve tendon healing. The effect of the treatment is dependent on the dose and timing of drug administration. We here suggest that the drug ought to be administered during the proliferative or early remodeling phase for an optimal outcome. The therapeutic effect of dexamethasone is apparent even with moderate loading, which relates more to the human situation, where full loading is avoided. Thus, the findings indicate a promising prospect for improving the material properties of the healing tendon with the use of delayed dexamethasone treatment.

## Supplemental Material

sj-pdf-1-ajs-10.1177_03635465221077101 – Supplemental material for Dexamethasone Enhances Achilles Tendon Healing in an Animal Injury Model, and the Effects Are Dependent on Dose, Administration Time, and Mechanical Loading Stimulation Click here for additional data file. Supplemental material, sj-pdf-1-ajs-10.1177_03635465221077101 for Dexamethasone Enhances Achilles Tendon Healing in an Animal Injury Model, and the Effects Are Dependent on Dose, Administration Time, and Mechanical Loading Stimulation by Franciele Dietrich-Zagonel, Per Aspenberg and Pernilla Eliasson in The American Journal of Sports Medicine
