# Supplementary material for: Dexamethasone Enhances Achilles Tendon Healing in an Animal Injury Model, and the Effects Are Dependent on Dose, Administration Time, and Mechanical Loading Stimulation
Source: Am J Sports Med. 2022 Mar 2;50(5):1306–16. doi: 10.1177/03635465221077101 (PMC9014685; doi:10.1177/03635465221077101)
Supplement: sj-pdf-1-ajs-10.1177_03635465221077101 – Supplemental material for Dexamethasone Enhances Achilles Tendon Healing in an Animal Injury Model, and the Effects Are Dependent on Dose, Administration Time, and Mechanical Loading Stimulation [file sj-pdf-1-ajs-10.1177_03635465221077101.pdf]

Dexamethasone enhances Achilles tendon healing in an animal injury model and the effects are dependent on dose, administration time, and mechanical loading stimulation.

Appendix

Table A1. **Antibodies specificities and flow cytometer setup.** Cat# means Catalogue Number.

Antigen	Fluorochrome	Clone	Titer	Stock conc. (g/l)	Mirror:Filter	Supplier	CAT#
CD45	Pe-Cy7	OX-1	1:1500	0.2	735LP:780/60	Biolegend	202214
CD11b	V500	M1/70	1:200	0.2	502LP: 510/50 BP	BD	562127
CD3	AF647	1F4	1:2000	0.5	-:660/20	Biolegend	201408
CD4	PE	OX-35	1:2000	0.2	-:582/15	Biolegend	203307
CD8a	PerCP	OX-8	1:200	0.2	502LP:675/20	Biolegend	201712

Table A2: Mechanical results from Experiment 1. Percentage was calculated in relation of the saline group. n = 10 for each group (both experiments), except for Dexa 0.1mg/kg (n=9) in experiment 1 (7am). SD means standard deviation, Dexa means dexamethasone and Est. elastic modulus means estimation of elastic modulus. Bold means significant difference compared to saline.

	Saline Mean (SD)	Dexa 5x 0.5mg/kg Mean (SD)	P Value	%	Dexa 5x 0.1mg/kg Mean (SD)	P Value	%	Dexa 1x 0.5mg/kg Mean (SD)	P Value	%	Dexa 2x 0.5mg/kg Mean (SD)	P Value	%
Experiment 1 (11am)													
Material properties													
Peak stress, MPa	3.0 (0.7)	3.8 (0.5)	**0.008**	27	3.7 (0.5)	**0.026**	23	3.4 (0.8)	0.245	13	-	-	-
Est. elastic modulus, MPa	3.3 (1.0)	5.6 (0.8)	**<0.001**	70	5.3 (1.4)	**0.002**	61	4.7(1.5)	**0.032**	42	-	-	-
Structural properties													
Transverse area, mm^2	15 (2.8)	10.6 (2.2)	**0.001**	-29	11.6 (2.1)	**0.010**	-23	12 (3.2)	0.062	-20	-	-	-
Gap length, mm	8 (1.0)	9.7 (1.5)	**0.008**	21	9.3 (0.8)	**0.005**	16	9.3 (1.4)	**0.029**	16	-	-	-
Peak force, N	45 (11)	41 (12)	0.464	-9	43 (10)	0.689	-4	40 (8)	0.321	-11	-	-	-
Stiffness, N/mm	6 (1.1)	6.2 (1.5)	0.679	3	6.4 (0.9)	0.372	7	5.9 (1.1)	0.807	-2	-	-	-
Energy uptake, N/mm	111 (46)	79 (33)	0.087	-29	93 (26)	0.294	-16	87 (22)	0.154	-22	-	-	-
Repetition of experiment 1 (7am)													
Material properties													
Peak stress, MPa	3.0 (0.5)	4.2 (1)	**0.004**	40	3.2 (0.9)	0.582	7	2.9 (0.6)	0.620	-3	3.0 (0.6)	0.957	0
Est. elastic modulus, MPa	3.7 (1.1)	7.5 (2)	**<0.001**	103	4.7 (1.3)	0.060	27	3.6 (1.1)	0.897	-3	4.2 (0.8)	0.252	14
Structural properties													
Transverse area, mm^2	13 (2.8)	8.9 (1.6)	**<0.001**	-32	11 (2.7)	**0.042**	-15	13 (2.6)	0.666	0	12 (1.5)	0.107	-8
Gap length, mm	7.8 (1.4)	10 (0.9)	**<0.001**	28	8.8 (1)	0.086	13	8.3 (0.9)	0.370	6	8.6 (0.8)	0.117	10
Peak force, N	40 (9)	36 (5.1)	0.298	-10	32 (7.1)	0.063	-20	36 (8.5)	0.405	-10	35 (7.1)	0.200	-13
Stiffness, N/mm	6.1 (1.8)	6.5 (1.2)	0.625	7	5.4 (0.8)	0.269	-11	5.4 (1.2)	0.287	-11	5.6 (1.2)	0.426	-8
Energy uptake, N/mm	91 (25)	68 (17)	**0.029**	-25	69 (21)	**0.043**	-24	93 (29)	0.838	2	83 (14)	0.392	-9
Sample weight, g	2.1 (0.3)	1.6 (0.3)	**0.001**	-24	2 (0.3)	0.172	-5	2.1 (0.3)	0.932	0	2.0 (0.2)	0.215	-5

Table A3: Mechanical results from Experiment 5. Percentage was calculated in relation of the saline group (n = 10 in each group). SD means standard deviation, Dexa means dexamethasone and Est. elastic modulus means estimation of elastic modulus. Bold means significant difference compared to saline, while * means significant difference compared to dexamethasone.

	Saline Mean (SD)	Dexa Mean (SD)	P Value	%	Late mod. loading Mean (SD)	P Value	%
Material properties							
Peak stress, MPa	2.6 (0.7)	4.0 (1.2)	**0.004**	54	1.7 (0.7)*	0.098	-35
Est. elastic modulus, MPa	3.9 (1.3)	5.9 (2.6)	0.100	51	2.8 (1.8)*	0.771	-28
Structural properties							
Transverse area, mm^2	12 (3.1)	9.2 (2.0)	0.086	-23	7.0 (1.7)	**<0.001**	-42
Gap length, mm	8.3 (1.0)	7.9 (1.6)	>0.999	-5	6.8 (1.1)	**0.049**	-18
Peak force, N	29 (9.4)	35 (7.2)	0.227	21	11 (4.6)*	**<0.001**	-62
Stiffness, N/mm	5.1 (1.5)	6.4 (1.4)	0.204	25	2.8 (1.6)*	**0.006**	-45
Energy uptake, N/mm	69 (20)	71(15)	>0.999	3	24 (6.4)*	**<0.001**	-65
Sample weight, g	2.1 (0.3)	1.8 (0.2)	0.066	-14	1.6 (0.2)	**<0.001**	-24

Table A4: Flow cytometry data from experiment 4. Rats received dexamethasone (systemic or local injections) or saline (systemic) during day 7-11. Percentage was calculated in relation of the saline group (n=6 in each group except for local injections, n=2). SD means standard deviation and Dexa means dexamethasone. Bold number means significant difference compared to saline, while * means significant different comparing to dexamethasone injected systemically.

	Saline Systemic Mean (SD)	Dexa Systemic Mean (SD)	P value	%	Dexa Local Mean (SD)	P value	%
Granulocytes	0.07 (0.05)	0.22 (0.05)	**<0.001**	214	0.35 (0.06)*	**<0.001**	400
CD11B	0.05 (0.02)	0.18 (0.05)	**<0.001**	260	0.33 (0.05)*	**<0.001**	560
Lymphocytes	0.86 (0.05)	0.68 (0.04)	**<0.001**	-21	0.56 (0.04)*	**<0.001**	-35
T cells	0.45 (0.04)	0.27 (0.07)	**<0.001**	-40	0.21 (0.01)	**<0.001**	-53
CD4	0.27 (0.03)	0.18 (0.05)	**0.003**	-33	0.14 (0.01)	**0.001**	-48
CD8	0.16 (0.03)	0.08 (0.02)	**0.001**	-50	0.06 (0.01)	**0.008**	-63
